# CD26-positive/CD326-negative circulating cancer cells as prognostic markers for colorectal cancer recurrence

**DOI:** 10.3892/ol.2014.2749

**Published:** 2014-12-01

**Authors:** EVA LIETO, GENNARO GALIZIA, MICHELE ORDITURA, CIRO ROMANO, ANNA ZAMBOLI, PAOLO CASTELLANO, ANDREA MABILIA, ANNAMARIA AURICCHIO, FERDINANDO DE VITA, MARICA GEMEI

**Affiliations:** 1 Division of Surgical Oncology, Department of Anesthesiological, Surgical and Emergency Sciences, Second University of Naples School of Medicine, Naples I-80131, Italy; 2 Division of Medical Oncology, ‘F. Magrassi-A. Lanzara’ Department of Clinical and Experimental Medicine and Surgery, Second University of Naples School of Medicine, Naples I-80131, Italy; 3 Division of Internal Medicine, Allergy and Clinical Immunology, Department of Medical and Surgical Sciences, Second University of Naples School of Medicine, Naples I-80131, Italy; 4 Center for Genetic Engineering, Advanced Biotechnologies, Naples I-80145, Italy

**Keywords:** cluster of differentiation 26, circulating tumor cell, patient management, colorectal cancer, flow cytometry

## Abstract

The present study evaluated the presence and clinical relevance of a cluster of differentiation (CD)26^+^/CD326^−^ subset of circulating tumor cells (CTCs) in pre- and post-operative blood samples of colorectal cancer patients, who had undergone curative or palliative intervention, in order to find a novel prognostic factor for patient management and follow-up. In total, 80 colorectal cancer patients, along with 25 healthy volunteers were included. The easily transferable methodology of flow cytometry, along with multiparametric antibody staining were used to selectively evaluate CD26^+^/CD326^−^ CTCs in the peripheral blood samples of colorectal cancer patients. The multiparametric selection allowed any enrichment methods to be avoided thus rendering the whole procedure suitable for clinical routine. The presence of CD26^+^/CD326^−^ cells was higher in advanced Dukes’ stages and was significantly associated with poor survival and high recurrence rates. Relapsing and non-surviving patients showed the highest number of CD26^+^/CD326^−^ CTCs. High pre-operative levels of CD26^+^/CD326^−^ CTCs correctly predicted tumor relapse in 44.4% of the cases, while 69% of post-operative CD26^+^/CD326^−^ CTC-positive patients experienced cancer recurrence, with a test accuracy of 88.8%. By contrast, post-operative CD26^+^/CD326^−^ CTC-negative patients showed an increase in the three-year progression-free survival rate of 86%, along with a reduced risk of tumor relapse of >90%. In conclusion, CD26^+^/CD326^−^ CTCs are an independent prognostic factor for tumor recurrence rate in multivariate analysis, suggesting that their evaluation could be an additional factor for colorectal cancer recurrence risk evaluation in patient management.

## Introduction

Despite advances in the surgical and therapeutic treatment of colorectal cancer patients, ~30–50% of patients develop tumor relapse and metastases (1). It is known that colonic adenomas and cancerous lesions easily release cells at a percentage rate five times higher than that of the normal mucosa (2,3). Due to the malignant characteristics of a number of the released cancer cells, they can easily enter the blood circulation and migrate to distant organ sites. This cell dissemination has been recently demonstrated to be an early event, even if only weakly present in initial stages of cancer patients (4,5). Circulating tumor cells (CTCs) may not only establish metastases in distant organs, but may also self-seed back to their origin organ, promoting tumor relapse (6). The detection of CTCs has been associated with a poor prognosis and poor survival rate of colorectal cancer patients (7–9). However, a number of problems affect CTC identification and enumeration; each method used for their detection has several drawbacks (10–13). The scarcity of CTCs in peripheral blood samples means that an enrichment step is often required prior to the analysis (14,15), however, all methods used to enrich CTCs from blood samples (i.e., filtration, density gradient separation and magnetic isolation) exhibit a low purity grade (16–18).

Furthermore, major techniques used to identify CTCs (i.e., quantitative polymerase chain reaction and CellSearch system) are too expensive and time consuming to be predominantly used in the clinical routine (10,19). In addition, nucleic acid-based methods are markedly affected by the presence of a huge number of contaminant cells inside peripheral blood samples (9,12), while intact cell analysis by the use of specific markers is defective due to the lack of unambiguous specificities (9,12,14); in fact several of the markers expressed by cancer cells are shared by leucocytes. Flow cytometry is a suitable technique to analyze CTCs (19–22), as it allows single cell analysis and permits the researcher to include or exclude doubtful origin populations and suspect objects from the analysis at any time following sample acquisition. The universally recognized markers of CTCs are the epithelial specificities, CD326 (EpCAM) and the cytokeratins (9,23,24), however, recent findings have highlighted the complex nature of cancer cell dissemination, which involves deep cell changes, including the epithelial-to-mesenchymal (EMT) transition (25–30). The types of modifications that occur in the cell in such transitions are not univocally clarified; it has been demonstrated that cells reduce the epithelial characteristics as mesenchymal features appear (25,26), this transition appears to promote cancer cell dissemination (31–33). In addition, it has been reported that intermediate phenotypes are observable in CTCs with the presence of epithelial and mesenchymal markers (25,33,34). The assessment of biologically significant markers is likely to provide more clinically relevant information than simple enumeration (35), therefore, the development of a precise, accurate and reproducible assay to detect the CTC level in the peripheral blood of colorectal cancer patients requires evaluation of the different cancer cell populations that possess the ability to enter the blood circulation. Therefore, the current study selected two markers to explore the CTC populations in the peripheral blood of colorectal cancer patients by flow cytometry. The aim of the study was to investigate the commonly used epithelial specificity, CD326/EpCAM, and the metastatic colon cancer cell marker, CD26/DPPIV (36–38).

## Materials and methods

### Patients

Patients observed between September 2009 and August 2012 at the Second University of Naples School of Medicine (Naples, Italy) with histologically confirmed colorectal adenocarcinoma were considered eligible for this study. Patients meeting the Amsterdam II criteria (39) for hereditary non-polyposis colorectal cancer syndrome or with carcinomas associated with inflammatory bowel disease were excluded from the study. In total, 80 patients, consisting of 48 colon and 32 rectal cancer patients, were included in the study. In addition, 25 healthy subjects served as a control. Informed consent was obtained for each patient or healthy volunteer. According to the pre-operative staging utilizing echo endoscopy and pelvic magnetic resonance imaging, 14 (43.8%) rectal cancer patients underwent pre-operative chemoradiotherapy (CRT) with a 5-fluorouracil- and oxaliplatin-based regimen and a total radiation dose of 45 Gy, followed by surgery at approximately six to eight weeks following completion of the CRT. Furthermore, 72 (90.0%) patients, including 14 with liver-limited metastatic tumors, underwent potentially curative resection (R0 resection), defined as the removal of all of the macroscopic tumoral masses, the absence of microscopic residual tumor, histology-negative resection margins and negative peritoneal cytology (40). In the remaining eight patients, including six patients with distant metastases, the primary tumor was resected, however, the surgery was determined to be non-curative due to local or distant diffusion.

### Follow-up

All surgeries were successful and without perioperative mortality. All patients were discharged from the hospital and underwent adjuvant chemotherapy if appropriate. In the rectal cancer cases, adjuvant chemotherapy was performed for pT3 tumors, as well as in the patients treated with neoadjuvant CRT, regardless of post-operative pathological staging (41). Follow-up included a physical examination and carcinoembryonic antigen (CEA) serum level analysis every three months for the first two years, colonoscopy at the one year mark and liver ultrasonography every six months for two years. Chest/abdominal/pelvic computed tomography (CT) scans were performed annually; those patients undergoing liver resection entered a more strict follow-up, including liver ultrasonography every three months and CT scan every six months. In cases where recurrence was suspected, patients were subjected to further diagnostic methods always complemented by routine histopathological examination of a biopsy specimen. In total, 18 of the selected patients experienced cancer relapse.

The following clinicopathological parameters were recorded: Age, gender, tumor site (right and left colon, and rectum), serum CEA levels, performance status according to the Eastern Cooperative Oncology Group scale (39), neoadjuvant CRT, post-operative chemotherapy and complication rate, tumor size, tumor-node-metastasis staging system (42) and Dukes’ stage, number of harvested nodes and positive nodes, lymph node ratio (LNR; the ratio between positive nodes and resected nodes), histological differentiation (well, moderate or poor), oncological radicality, recurrence and progression-free survival (PFS) rates.

No patient was lost to follow-up, which was completed by February 28, 2012. All patients provided written informed consent and the study was approved by the Division of Surgical Oncology, Department of Anesthesiological, Surgical and Emergency Sciences of the Second University of Naples School of Medicine (Naples, Italy).

### Endpoints

Primary endpoints were the actual capacity of the method to evaluate the levels of CD26^+^/CD326^−^ cells in the peripheral blood, post-operative modifications of these levels and their correlations with outcome. PFS rate was used to investigate the correlation between the blood levels of CD26^+^/CD326^−^ cells and tumor recurrence. Finally, the independent significance of CD26^+^/CD326^−^ cells in predicting cancer relapse was evaluated by multivariate analysis. Secondary endpoints were the correlation between the blood levels of CD26^+^/CD326^−^ cells and other prognostic factors, particularly tumor radical resection, pre-operative therapy and tumor stage.

### Statistical analysis

Statistical analysis was performed using the SPSS statistical package (SPSS Inc., Chicago, IL, USA) integrated by MedCalc^®^ software, version 9.4.2.0 (MedCalc, Mariakerke, Belgium). In all analyses, the significance level was specified as P<0.05. Data are presented as the mean ± standard deviation*,* range and median values. The equality of group means and comparisons between proportions were analyzed using a paired Student’s t*-*test and χ^2^ test, respectively.

Linear regression was used to investigate the correlations among different blood levels of CD26^+^/CD326^−^ cells and other prognostic factors. Univariate analysis associated with PFS was limited to radically resected patients and determined by log-rank test (Mantel-Cox). Curves were plotted using the Kaplan-Meier method, providing the P-value and hazard ratio with a 95% confidence interval (CI). The independent significance of the prognostic variables was determined by multivariate analysis, using Cox’s proportional hazards model.

### Peripheral blood sample treatment

For each patient and healthy control in the study, 7.5 ml of peripheral blood was harvested and diluted 1:2 with phosphate-buffered saline (PBS; Sigma-Aldrich, St. Louis, MO, USA) without Ca^2+^/Mg^2+^. Mononucleated cells were recovered from peripheral blood samples by centrifugation using a Ficoll Histopaque (Sigma-Aldrich) for 30 min at 320 × g. The mononuclear cell ring was isolated and washed twice with PBS at 320 × g for 3 min. All recovered cells were stained with the appropriate amount of mouse anti-human monoclonal anti-CD326 PerCP-Cy5.5, anti-CD45-APC-Cy7 and anti-CD26^−^FITC antibodies (BD Bioscience, San Jose, CA, USA). For each patient, a pre- and post-operative peripheral blood sample (7.5 ml each) was analyzed. The post-operative sample was obtained one month after the surgical treatment. In addition, 25 samples obtained from the healthy controls were used as control.

## Results

### Identification of a CD26^+^/CD326^−^ CTC population and comparison with CD326^+^ CTCs present in colorectal cancer patients

**Figure 1:**
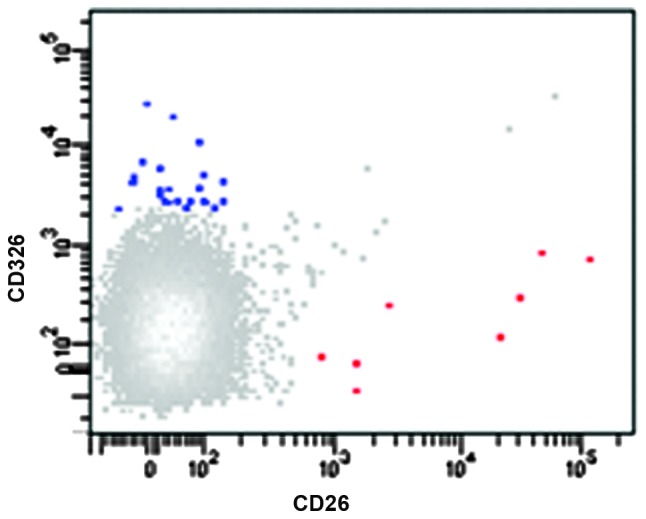
CD326^+^ and CD26^+^/CD326^−^ CTC detection. The CD326 versus CD26 dot plot shows a representative pre-operative sample of peripheral blood in which CD326^+^ (blue) and CD26^+^/CD326^−^ (red) CTCs were detected. Cells were identified in the CD45-negative non-hematopoietic cell population. CD, cluster of differentiation; CTC, circulating tumor cell.

A multiparametric cytometric analysis was used to evaluate the CD26^+^/CD326^−^ CTCs present in the peripheral blood samples of the colon cancer patients and healthy controls. The cells were first gated on physical parameters in a dot plot [forward scatter versus side scatter (SSC)] to exclude debris. Subsequently, in a CD45 versus SSC dot plot, the CD45^−^ cell population position was identified by discarding all hematopoietic contaminants. Finally, in a CD326 versus CD26 dot plot, ‘conventional’ CD326^+^ and CD26^+^/CD326^−^ CTCs were identified (Fig. 1). Furthermore, the expression of three colorectal cancer stem cell markers, CD44, CD66c and CD133, were evaluated (43,44) on CD326^+^ and CD26^+^/CD326^−^ cells, observing the consistent expression of CD44 and CD66c along with a substantial negativity for CD133 (data not shown). CD326^+^ and CD26^+^/CD326^−^ cells were identified in the peripheral blood samples of the 25 healthy controls and 80 colorectal cancer (60 non-metastatic and 20 metastatic) patients. Analysis of the healthy controls allowed a cut-off to be defined in order to determine the background level for the analysis. In the healthy subjects, at least one CD326^+^ CTC was counted, however, no CD26^+^/CD326^−^ cells were identified. Therefore, positive patients samples were considered to exhibit ≥1 CD326^+^ and >0 CD26^+^/CD326^−^ CTCs. CD326^+^ tumor cell circulation was found to be a more common event than CD26^+^/CD326^−^ cell spreading; 62 patients were pre-operatively positive for CD326^+^ cells, 36 of whom were also positive for CD26^+^/CD326^−^ cells. Furthermore, the pre-operative blood levels of CD326^+^ cells in the cancer patients ranged between 0 and 165 (mean, 24.45±34.92; 95% CI, 12.97–35.92; median, 11.50), while those of the CD26^+^/CD326^−^ cells ranged between 0 and 24 (mean, 3.97±6.4; 95% CI, 2.53–5.41; median, 0). CD326^+^ cell spreading is likely to be the first event in cancer cell dissemination, since no patients were positive for CD26^+^/CD326^−^ cells, but they were positive for CD326^+^ cells. Pre-operative CD326^+^ cells were identified in 48 M0 and 14 M1 patients. All patients who experienced cancer relapse exhibited pre-operative CD326^+^ cells, while CD26^+^ cells were detected in 26 M0 and 10 M1 patients pre-operatively. All but two patients who experienced cancer recurrence were positive for CD26^+^/CD326^−^ cells pre-operatively. In the post-operative analysis, eight M0 and 10 M1 patients were CD326^+^ CTC-positive, while 16 M0 and 10 M1 were positive for CD26^+^/CD326^−^ cells. Post-operative CD326^+^ was detected in the 66.6% of patients who relapsed (12 out of 18), while CD26^+^/CD326^−^ cells predicted all relapses. No pre-operative CD326^+^ CTC-negative patients became positive post-operatively, while six patients who were CD26^+^/CD326^−^ CTC-negative pre-operatively became positive post-operatively; two experienced cancer relapse. The clinical relevance of CD326^+^ CTCs was analyzed in a previous study (45), therefore, now only CD26^+^/CD326^−^ cells are considered to correlate with the clinical outcome of colorectal cancer patients.

### Pre-operative analysis of CD26^+^/CD326^−^ CTCs

In Table I, the correlations between the blood levels of CD26^+^/CD326^−^ cells and other clinicopathological variables are shown. High pre-operative levels of CD26^+^/CD326^−^ cells tended to be more frequent in patients with advanced Dukes’ stages, and were significantly associated with the requirement for adjuvant chemotherapy, undifferentiated tumors, and poor recurrence and survival rates. Neoadjuvant CRT did not affect the blood levels of CD26^+^/CD326^−^ cells. Linear regression showed that the blood levels of CD26^+^/CD326^−^ cells were increased in younger patients with rectal cancer, high CEA levels, advanced Dukes’ stages and undifferentiated tumors. Relapsing and non-surviving patients showed the highest pre-operative levels of CD26^+^/CD326^−^ cells (r=0.475, P<0.001 for CD26^+^; and r=0.4237, P<0.001 for CD326^−^).

### Post-operative evaluation of CD26^+^/CD326^−^ CTCs

One month after surgery, the blood levels of CD26^+^/CD326^−^ cell levels had dropped significantly (mean, 1.20±2.36; 95% CI, 0.67–1.72; range, 0 to 11; median, 0; P<0.0001) compared with the pre-operative levels. Among the pre-operative CD26^+^/CD326^−^ CTC-negative patients, 38 remained negative and six (16%) showed post-operative CD26^+^/CD326^−^ positivity; in the pre-operative CD26^+^/CD326^−^ CTC positive group, 14 (38.9%) patients were post-operatively CD26^+^/CD326^−^ CTC-negative and 22 remained positive. Notably, among the 72 patients undergoing potentially curative surgery, 26 (36.1%) continued to show elevated post-operative blood levels of CD26^+^/CD326^−^ cells; by contrast, from the eight patients undergoing non-radical surgery, six (75.0%) showed normalized levels of CD26^+^/CD326^−^ cells, and two were CD26^+^/CD326^−^ CTC-positive. This suggested that post-operative blood levels of CD26^+^/CD326^−^ cells do not correlate with surgical and/or pathological radicality (r=0.1136, P=0.3162). Therefore, cancers that have supposedly been radically resected may continue to spread through the blood.

### Analysis associated with outcome

**Figure 2:**
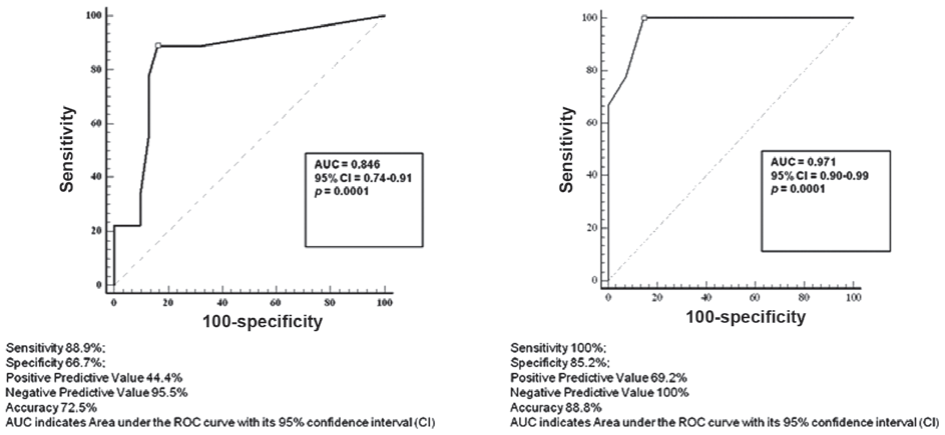
ROC curve analysis at different values of (A) pre- and (B) post-operative blood levels of CD26^+^/CD326^−^ cells is shown. The value of 0 cells for 7.5 ml of peripheral blood is indicated with a square sign and corresponds with the highest accuracy (minimal false-negative and false-positive results). ROC, receiver operating characteristic; AUC, area under curve; CI, confidence interval; CD, cluster of differentiation.

**Figure 3:**
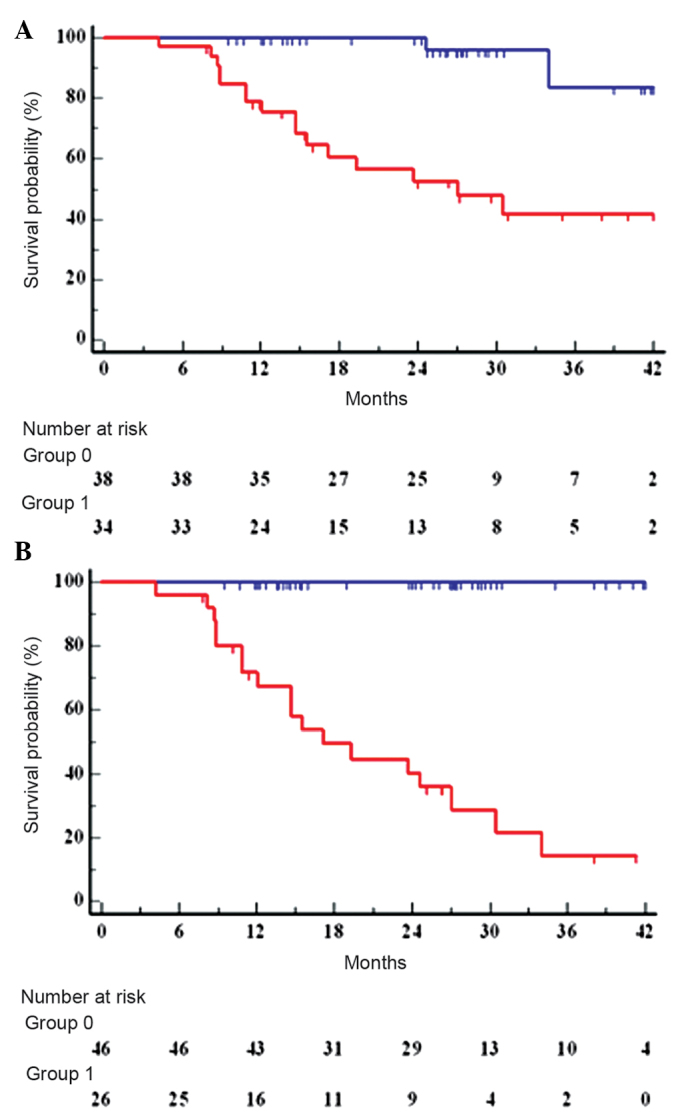
Three-year progression-free survival rate in the 72 colorectal cancer patients who underwent potentially curative surgery according to (A) pre- and (B) post-operative blood levels of CD26^+^/CD326^−^ cells. Group 0, CD26^+^/CD326^−^ cell-negative patients and group 1, CD26^+^/CD326^−^ cell-positive patients.

The mean follow-up time was 23±10 months (95% CI, 21–25; range, 3–42; median, 25). In this time period, 10 patients succumbed to cancer; the one- to three-year overall survival rates were 96.3, 85.8 and 82.2%, respectively. All but two patients who succumbed showed high pre- and post-operative levels of CD26^+^/CD326^−^ cells. Among the 72 patients undergoing potentially curative surgery, 18 (25%) patients showed tumor recurrence (mean, 16±8; 95% CI, 12–20; range, 4–34; median, 15 months). The one- to three-year overall PFS rates were 90.0, 77.2 and 63.3%, respectively. No tumor recurrence was experienced after 36 months. High pre-operative blood levels of CD26^+^/CD326^−^ cells correctly predicted tumor relapse in 44.4% of the cases (Fig. 2A); by contrast, patients with normal pre-operative blood levels of CD26^+^/CD326^−^ cells developed recurrence in only 5% of cases (test accuracy, 72.5%). The performance of the test of post-operative blood levels of CD26^+^/CD326^−^ cells in predicting tumor recurrence was even more impressive, with a test accuracy of 88.8% (Fig. 2B). Among the 26 patients showing high post-operative blood levels of CD26^+^/CD326^−^ cells, 18 (69.2%) relapsed, while no tumor recurrence was observed among the 46 patients with normal post-operative blood levels of CD26^+^/CD326^−^ cells. A younger age, elevated serum CEA level, adjuvant chemotherapy, advanced pT stage, lymph node-positive status, distant metastases, advanced Dukes’ stage and limited lymph node yield were shown to significantly correlate with an increased recurrence rate. Poor performance status and high LNR were marginally associated with a poor outcome. Pre- and post-operative blood levels of CD26^+^/CD326^−^ cells were found to markedly correlate with the tumor relapse rate (Table II). Pre- and post-operative CD26^+^/CD326^−^ CTC-negative patients showed an increase in the three-year PFS rate of 42 and 86%, respectively, with a reduction of the relative risk of tumor relapse to >90% (Fig. 3). Together with the presence of distant metastases and advanced Dukes’ stage, pre- and post-operative high blood levels of CD26^+^/CD326^−^ cells were found to be independent prognostic factors that correlated with an increased tumor recurrence rate on multivariate analysis (Table III). The statistical power of the described variables covered the prognostic significance of factors known to affect long-term outcome, including pT and pN status. Pre- and post-operative high blood levels of CD26^+^/CD326^−^ cells conditioned a probability of tumor relapse that was, respectively, 11 and 18 times higher than that observed in patients who had no detectable blood levels of CD26^+^/CD326^−^ cells.

## Discussion

One of the most important drawbacks for CTC detection and its usefulness in the clinic is the lack of specific markers of biologically important subsets of CTCs (19,23,35,46,47). CD326 has been confirmed to be adequate to isolate CTCs (18,23), even if its use has been recently questioned, as circulating CD326^+^ cells are also detected in patients with benign diseases of the colon (21). Furthermore, in colon cancer, EMT and the loss of epithelial specificities has been observed in cells that are prone to entering the blood circulation (26,27,30,31). The present study analyzed the expression of the epithelial marker, CD326/EpCAM, and the metastatic cancer cell marker, CD26/DPPIV (36) in CTCs obtained from colorectal cancer patients. The presence of two subsets of CTCs were demonstrated, one expressing CD326 and one expressing CD26, but each CD326-negative. CD26-positive cells from colon cancer exhibit metastatic abilities (36), and the results from the current study demonstrated, for the first time, their presence in the blood circulation of colorectal cancer patients and the correlation with patient prognosis for cancer recurrence. Furthermore, the expression of CD44, CD133 and CD66c protein expression was analyzed on the surface of the CTCs (data not shown), as they are described as colon cancer stem cell markers (43,44), and it is likely that these cells are more prone to entering the blood circulation to disseminate in the whole body and generate metastasis (27). The phenotypic analysis revealed that, in contrast to CD133, CD44, CD66c and CD26, are reliable markers of CTCs in colon cancer patients (data not shown). Notably, CD44 and CD66c showed functional properties in colon cancer cells, however, by contrast, the functional importance of CD133 appeared to be marginal (48–50). These results suggested that stem-like tumor initiating-cells are responsible for cancer spread and recurrence. In this study, relapsing patients showed a positivity for CD326^+^ and CD26^+^/CD326^−^ CTCs even when the primary cancer had a low pathological grade, suggesting that CTCs can identify high-risk colorectal cancer patients at early stages. Notably, the spread of CD326^+^ cancer cells into the blood circulation is a common event and 77.5% of all analyzed patients exhibited these cells in their peripheral blood pre-operatively, while 45.0% exhibited CD26^+^/CD326^−^ cells. Notably, only 50.0% of metastatic patients had CD26^+^/CD326^−^ cells in their bloodstream. This suggested that cancer cell spreading is a changeable process, which could be enhanced to favor cancer cell dissemination while being reduced once cancer cells have been reseeded in distant organs. As predicted, patients with high-grade and advanced stage cancers showed a higher positivity for CD26^+^/CD326^−^ CTCs. Furthermore, relapsing and non-surviving patients had the highest positivity observed. CD26^+^/CD326^−^ cell number was increased in young patients, suggesting that the spread of the cancer cells is slowed by increasing age. Curative surgery was confirmed to be fundamental for CD26^+^/CD326^−^ CTC reduction, even if a number of pre-operative CD26^+^/CD326^−^ CTC-positive patients continued to exhibit CTCs following surgery. This demonstrated that CTCs have the ability to survive for a long time, however, advanced studies are required to assess if CTCs survive in the bloodstream or in a niche, such as the bone marrow (51,52). Post-operatively, 22 patients (61.1%) remained positive for CD26^+^/CD326^−^ CTCs, while six patients (16%) who were pre-operatively negative became positive. The limited effect of surgery in the elimination of these CTCs suggested that CD26^+^/CD326^−^ cells function as the root of the tumor and are probably induced to circulate for tumor growth or expansion. Overall, the results of the current study suggested that CD26^+^/CD326^−^ cells are likely to be responsible for metastatic processes and cancer recurrence. Therefore, their identification may have clinical relevance in the evaluation of risk assessment for patients. Patients with pre- and/or post-operative CD26^+^/CD326^−^ CTCs had an 11- to 18-times higher risk of cancer recurrence. Further studies are required to investigate the biological function of this cancer cell subset, while a refined assay for their detection may provide an incentive to their use in the clinical management of colorectal cancer patients.

## Figures and Tables

**Table I:** Clinicopathological characteristics of the series, and correlations between pre-operative blood levels of CD26^+^/CD326^−^ cells and other factors.

Variables	CD26^+^/CD326^−^-negative (n=44)	CD26^+^/CD326^−^-positive (n=36)	P-value^a^
Age, years^b^			
≤66	22	20	0.7871
>66	22	16	
Gender			
Male	30	22	0.6715
Female	14	14	
Tumor site			
Right colon	6	8	0.2936
Left colon	22	12	
Rectum	16	16	
Serum CEA levels, ng/ml^c^			
≤3.5	30	18	0.1550
>3.5	14	18	
Performance status^d^			
0	22	18	0.1857
1	14	16	
2	8	2	
Neoadjuvant CRT^e^			
No	10	8	0.7216
Yes	6	8	
Adjuvant chemotherapy			
No	16	4	0.0195
Yes	28	32	
Post-operative complications			
No	44	32	0.0796
Yes	0	4	
Tumor size, cm^b^			
≤4	24	22	0.7161
>4	20	14	
pT stage			
1	4	2	0.5343
2	12	6	
3	16	18	
4	12	10	
pN stage			
Lymph node-negative	24	14	0.2420
Lymph node-positive	20	22	
pM stage			
M0	34	26	0.7952
M1	10	10	
Dukes’ stage			
A	16	4	0.0676
B	8	8	
C	10	14	
D	10	10	
Histological differentiation			
Well	26	12	0.0018
Moderate	18	16	
Poor	0	8	
Radical resection			
Yes	38	34	0.4099
No	6	2	
Tumor recurrence			
No	42	20	0.0001
Yes	2	16	
Survival			
Yes	42	28	0.0001
No	2	8	

a χ^2^ test;

b median value;

c normal value in healthy subjects;

d performance status according to the Eastern Cooperative Oncology Group scale;

e only rectal cancer patients.

CEA, carcinoembryonic antigen; CRT, chemoradiotherapy; CD, cluster of differentiation.

**Table II:** Univariate analysis associated with PFS in 72 patients with colorectal cancer who underwent potentially curative surgery.

Variables	n	42-month PFS, %	Hazard ratio	Hazard ratio (95% CI)	P-value
Age ≤67/>67, years^a^	40/32	52/79	2.99	1.04–6.68	0.0411
Gender M/F	46/26	70/52	0.66	0.24–1.73	0.3929
Tumor site					0.6842
Right colon	14	61			
Left colon	28	57			
Rectum	30	67			
Serum CEA levels, ng/ml^b^					0.0054
≤3.5	46	84	0.27	0.09–0.66	
>3.5	26	36			
Performance status 0/1/2^c^	36/28/8	49/83/100			0.0917
Neoadjuvant CRT, no/yes	60/12	66/51	0.67	0.18–2.26	0.4910
Adjuvant chemotherapy, no/yes	20/52	100/54	0.00	0.08–0.69	0.0084
Post-operative complications, no/yes	68/4	64/37	0.32	0.01–1.59	0.1149
Tumor size ≤4/>4, cm^a^	46/26	60/71	1.00	0.37–2.68	0.9912
pT stage					0.1588
1	6	100			
2	18	82			
3	32	56			
4	16	68			
1–2/3–4	24/48	86/55	0.24	0.13–0.96	0.0428
pN stage, negative/positive	38/34	85/45	0.22	0.10–0.65	0.0043
pM stage, M0/M1	58/14	77/17	0.25	0.03–0.47	0.0017
Dukes’ stage					0.0012
A	20	100			
B	16	85			
C	22	56			
D	14	17			
A–B/C–D	36/36	93/54	0.11	0.07–0.46	0.0008
Harvested Nodes, n^a^					0.0391
<15	34	42	2.67	1.05–6.90	
>15	38	78			
Lymph Node Ratio^d^					0.0720
<0.1818	14	79	0.28	0.12–1.09	
>0.1818	20	31			
Histological differentiation					0.3026
Well	34	85			
Moderate	32	66			
Poor	6	56			
Pre-operative CD26^+^/CD326^−^ cells					<0.0001
Negative	38	84	0.08	0.05–0.35	
Positive	34	42			
Post-operative CD26^+^/CD326^−^ cells					
Negative	46	100	0.00	0.00–0.07	<0.0001
Positive	26	14			

a Median value;

b normal value in healthy subjects;

c Performance status according to the Eastern Cooperative Oncology Group scale;

d only lymph node-node positive patients and according to the cut-off value.

PFS, progression-free survival; CI, confidence interval; M, male; F, female; CEA, carcinoembryonic antigen; CRT, chemoradiotherapy.

**Table III:** Multivariate analysis associated with progression-free survival in 72 patients with colorectal cancer who underwent potentially curative surgery.

Variables	Coefficient	Standard error	Hazard ratio	Hazard ratio (95% CI)	P-value
Younger age	1.1577	1.6664	3.18	0.12–82.04	0.4872
Elevated serum CEA levels	0.6012	0.8281	1.82	0.36–9.17	0.4678
Adjuvant chemotherapy	0.8637	0.5977	2.37	0.73–7.60	0.1484
Advanced pT stage	1.1986	1.1068	3.31	0.38–28.69	0.2788
Lymph node-positive	1.3721	1.5026	3.94	0.21–73.85	0.3612
Distant metastases	2.1588	0.6452	8.66	2.46–30.47	0.0008
Advanced Dukes’ stage	1.8925	0.6936	6.63	1.71–25.66	0.0063
<15 harvested nodes	1.2570	1.4078	3.51	0.22–54.71	0.3719
High pre-operative levels of CD26^+^/CD326^−^ cells	2.4530	0.6506	11.62	3.26–41.33	0.0001
High post-operative levels of CD26^+^/CD326^−^ cells	2.8901	0.7769	17.99	3.92–82.50	0.0001

Multivariate analysis was performed including variables with significant value on univariate analysis, by Cox’s proportional hazards model. CI, confidence interval; CEA, carcinoembryonic antigen.
